# Intensity‐modulated radiotherapy versus proton radiotherapy versus carbon ion radiotherapy for spinal bone metastases: a treatment planning study

**DOI:** 10.1120/jacmp.v16i6.5618

**Published:** 2015-11-08

**Authors:** Harald Rief, Naved Chaudhri, Eric Tonndorf‐Martini, Thomas Bruckner, Stefan Rieken, Tilman Bostel, Robert Förster, Ingmar Schlampp, Jürgen Debus, Florian Sterzing

**Affiliations:** ^1^ University Hospital of Heidelberg Department of Radiation Oncology Heidelberg Germany; ^2^ University Hospital of Heidelberg Department of Medical Biometry Heidelberg Germany; ^3^ Heidelberg Ion Beams Therapy Center Heidelberg Germany; ^4^ Heidelberg Institute of Radiation Oncology (HIRO) Heidelberg Germany

**Keywords:** bone metastases, spine, carbon ion, proton radiotherapy, SBRT

## Abstract

Outcomes for selected patients with spinal metastases may be improved by dose escalation using stereotactic body radiotherapy (SBRT). As target geometry is complex, we compared SBRT plans using step‐and‐shoot intensity‐modulated radiotherapy (IMRT), carbon ion RT, and proton RT. We prepared plans treating cervical, thoracic, and lumbar metastases for three different techniques — IMRT, carbon ion, and proton plans — to deliver a median single 24 Gy fraction such that at least 90% of the planning target volume (PTV) received more than 18 Gy and were compared for PTV coverage, normal organ sparing, and estimated delivery time. PTV coverage did not show significant differences for the techniques, spinal cord dose sparing was lowered with the particle techniques. For the cervical lesion spinal cord maximum dose, dose of 1% (D1), and percent volume receiving 10 Gy ($V_{10\text{Gy}}$) were 11.9 Gy, 9.1 Gy, and 0.5% in IMRT. This could be lowered to 4.3 Gy, 2.5 Gy, and 0% in carbon ion planning and to 8.1 Gy, 6.1 Gy, and 0% in proton planning. Regarding the thoracic lesion no difference was found for the spinal cord. For the lumbar lesion maximum dose, D1 and percent volume receiving 5 Gy ($V_{5\text{Gy}}$) were 13.4 Gy, 8.9 Gy, and 8.9% for IMRT; 1.8 Gy, 0.7 Gy, and 0% for carbon ions; and 0 Gy,<0.01 Gy, and 0% for protons. Estimated mean treatment times were shorter in particle techniques (6–7 min vs. 12–14 min with IMRT). This planning study indicates that carbon ion and proton RT can deliver high‐quality PTV coverage for complex treatment volumes that surround the spinal cord.

PACS number: 87.55.dk

## I. INTRODUCTION

Spinal metastases are a common manifestation of malignant tumors, and progressive disease can result in pain, neurological disability, and reduced quality of life.^1^, ^2^ Radiosurgery can be used in primary or salvage treatment of spinal metastases, improving local disease control and patient symptoms.^3^, ^4^ Stereotactic body radiotherapy (SBRT) is being increasingly used in the management of localized spine metastases.^5^, ^6^, ^7^ The optimal treatment of individual patients with spinal metastases should include consideration of their neurologic status, anatomical extent of disease, general health, age, and quality of life.^1^, ^8^ The main challenge of spine SBRT is maintaining the cord dose within tolerance, as the planning target volume (PTV) is in close proximity to the cord. Spine radiosurgery permits a rapid dose falloff outside the target, and the most critical issues are related to the dose tolerance of the spinal cord.

Another important consideration for radiotherapy (RT) of the spine is the treatment time, as many patients experience substantial pain.^(9)^ Patients could benefit from hypofractionated or single‐fraction schedules.^10^, ^11^, ^12^ Outcome data show high rates of local control and pain control for spine metastases treated with SBRT, and suggest better efficacy than with conventional palliative radiotherapy.^11^, ^13^, ^14^

One important toxic effect is SBRT‐induced vertebral compression fracture with a crude risk ranging from 11% to 39%, which can be clinically significant, resulting in mechanical instability and a need for surgical intervention.^(15)^

We describe a planning study comparing IMRT, carbon ion RT and proton RT plans in single dose of 24 Gy for spinal SBRT. For the sake of simplicity and comparison we considered only physical doses for carbon ion and proton RT and, therefore, give the respective dose unit Gy only.

## II. MATERIALS AND METHODS

The planning CT scans of three patients previously treated for spinal metastases were included in a planning study to compare step‐and‐shoot IMRT, carbon ion scanned beam RT, and proton scanned beam RT. The techniques were planned using Oncentra MasterPlan by Nucletron (Veenendaal, The Netherlands) for IMRT, and TRiP98 for carbon ion and proton RT.^(16)^

Three cases were studied. Case 1 involved treatment to the cervical vertebral body (partial body and pedicle). Case 2 involved a thoracic lesion. Case 3 was in the lumbar spine. Spinal bone metastases are frequently located partially within the vertebral body with pedicle involvement, therefore the PTV was defined according to this critical location next to the cord. The PTV was 8.4 cc for case‐group 1, 30.2 cc for case‐group 2, and 54.7 cc for case‐group 3. Computed tomography (CT) and magnetic resonance imaging were used to diagnose spinal metastases. In each case, the radiosurgical treatment plan was designed based on tumor geometry, proximity to spinal cord, and location. First priority was sparing the spinal cord; covering tumor to RT dose was secondary.

The PTV was defined according consensus target volume definitions.^(17)^ A spinal cord planning volume at risk (PRV) was created by adding a 2 mm expansion to the contoured cord and/or cauda equina. The PTV was never expanded into this PRV. The PTV margin and a cord PRV reflected an overall treatment setup accuracy of approximately 2 mm for image‐guided spinal SBRT and to account for motion of the spinal cord^(18)^ and variation in the location of the cord within the thecal sac. The spinal cord was delineated on a planning CT scan after fusion with an MRI or CT myelography. The partial spinal cord and partial spinal cord PRV was limited to 6 mm above and below the PTV.^(19)^ Depending on the location of the vertebrae and the PTV, other organs at risk (OAR) that were segmented included liver, lungs, esophagus, bowel, skin, large vessels, and kidneys.

The prescription and planning objectives used for all techniques were the same. A single fraction of median 24 Gy was planned to the PTV such that at least 90% of the PTV received more than 18 Gy. Other objectives included a maximum PTV dose of 27 Gy, and a maximum point dose of 12 Gy to the spinal cord and 17 Gy to the partial cord PRV. OAR doses were aimed to be kept within suggested guidelines.^(6)^ The PTV was normalized to the same value for all plans of each case, although it could vary between cases to reflect differences in geometry of PTV and OAR. The conformity index was evaluated for the 24 Gy isodose (CI=total volume receiving 24 Gy (cc)/volume of PTV (cc), as is consistent with ICRU 62.^(20)^

### A. IMRT

IMRT plans for all groups were generated for an Elekta Synergy (Elekta AB, Stockholm, Sweden) linear accelerator with an integrated multileaf collimator (MLC) system. The configuration of nine static beams with 6 MV was used for each group. For PTV Dmax, Dmean, D95%, and the relative volume of the PTV receiving at least 24 Gy ($V_{24\text{Gy}}$) were assessed. For the spinal cord PRV, the Dmax and the relative volume receiving at least 12 Gy ($V_{12\text{Gy}}$) were assessed. Other OARs were evaluated according to the criteria described in the RTOG 0631 study.^(6)^ Beams on time were calculated for all plans using the total number of monitor units (MU) and assuming a dose rate of 600 MU/min to deliver treatment times of the three techniques.

### B. Carbon ion and proton RT

All the plans were generated for beam parameters used at our institute for patient treatment with scanned ion beams. Each case was planned with two dorsal beams. The beam angles were selected considering minimal range uncertainty due patient positioning and anatomical changes.

For all the three plans with carbon ion beams, beam spot size varied from 6–9 mm full width half maximum (FHWM) depending on the isoenergy layer, and step size in X and Y directions was 2 mm. The overlap in depth was 3 mm. The plans for proton beam were optimized with a spot size of 8–25 mm FHWM depending on the isoenergy layer. The step size in X and Y directions varied between 2 mm and 3 mm depending on the tumor size and site. Similarly, in depth the overlap varied between 2 mm and 3 mm.

The dose optimization method was multiple‐field optimization (i.e., intensity‐modulated particle therapy). DVH evaluation was performed with Slicer3D.^(21)^ DVH metrics explained in the IMRT section were calculated.

## III. RESULTS

**Figure 1:**
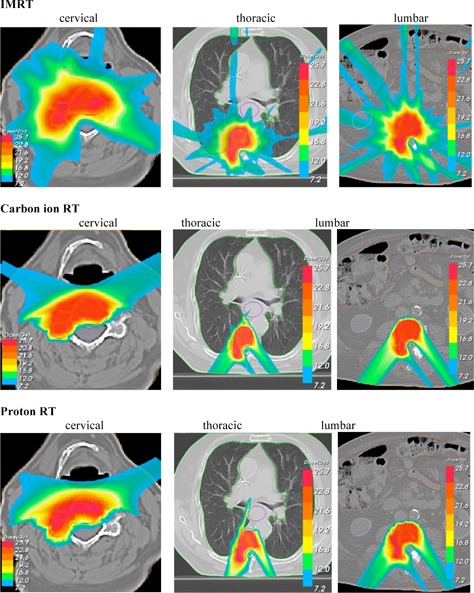
Dose distributions of IMRT, carbon ion, and proton plans in transverse view with isodose lines specified for 107%, 95%, 90%, 80%, 70%, 50%, and 30%.

**Figure 2:**
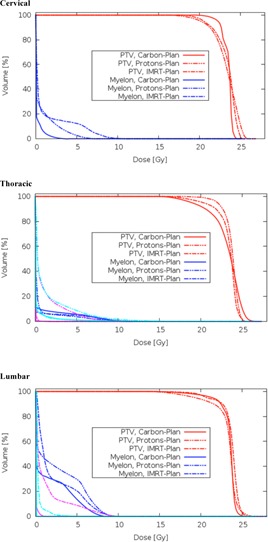
Dose‐volume histograms (DVHs) of IMRT, carbon ion beams, and proton beams in cervical, thoracic, and lumbar (PTV and myelon=spinal cord). X‐axis: Dose (Gy), y‐axis of ordinate: relative volume. Additional lines in "thoracic": light blue=lung both, magenta=lung right, additional lines in "lumbar": magenta=bowel.

With all three delivery techniques (IMRT, protons, and carbon ions) the planning objectives for spine SBRT could be met. Acceptable target coverage with more than 18 Gy at least 90% of the PTV and more than 23 Gy at mean dose, as well as dose along with the desired organ at risk sparing, were reached. Tables 1 to 3 display the dosimetric details of cervical, thoracic, and lumbar spine SBRT. Figures 1 and 2 show the corresponding dose distributions and DVHs.

While dose to the PTV did not show significant differences for the techniques, dose to the spinal cord was considerably lower with the particle techniques. For the cervical lesion spinal cord maximum dose, D1 and $V_{10\text{Gy}}$ were 11.9 Gy, 9.1 Gy, and 0.5%. This could be lowered to 4.3 Gy, 2.5 Gy, and 0% in carbon ion planning and to 8.1 Gy, 6.1 Gy, and 0% in proton planning. For the thoracic lesion no difference was found for the spinal cord. The D_10Gy_ of the esophagus was 5.6 Gy for photons and 1.0 Gy for carbon ions and 0.1 Gy for protons. Lung dose was significantly higher with photons with a $V_{5\text{Gy}}$ of 8.9 Gy (right lung) and 6.3 Gy (left lung), but still well within tolerance.

For the lumbar, lesion dose to the small bowel was considerably higher than with ions. Maximum dose, D1 and $V_{5\text{Gy}}$ were 13.4 Gy, 8.9 Gy, and 8.9% for photons; 1.8 Gy, 0.7 Gy and 0% for carbon ions; and 0 Gy,<0.01 Gy, and 0% for protons. The conformity index favored particle techniques in all vertebral segments: cervical 0.85, 1.29, and 1.10; thoracic 0.91, 1.01, and 1.31; and lumbar 0.93, 0.96, and 0.95 (IMRT, carbon ion, proton). The calculated time of irradiation for the plans with particle techniques ranged 6–7 min, and for the plans with IMRT, 12–14 min per plan.

**Table 1:** Dose calculation of cervical metastases and risk organs for IMRT, carbon ion RT, and proton RT

	*IMRT*					*Carbon ion*					*Proton*				
	*PTV*	*Myelon*	*PRV*	*Right Lung*	*Left Lung*	*PTV*	*Myelon*	*PRV*	*Right Lung*	*Left Lung*	*PTV*	*Myelon*	*PRV*	*Right Lung*	*Left Lung*
Volume (cc)	8.4	9.7	19.3	27.6	31.3	8.4	9.7	19.3	27.6	31.3	8.4	9.7	19.3	27.6	31.3
Dmean (Gy)	23.4	1.2	1.5	0.03	0.03	23.5	0.16	0.8	0.0	0.0	23.3	0.7	1.7	0.0	0.0
Drain (Gy)	17.1	0.03	0.03	0.02	0.02	17.7	0.0	0.0	0.0	0.0	15.4	0.0	0.0	0.0	0.0
Dmax (Gy)	25.4	11.9	13.16	0.06	0.05	24.7	4.3	18.4	0.0	0.0	26.3	8.1	16.9	0.0	0.0
V5 (%)	100.0	12.5	14.1	0.0	0.0	100.0	0.0	5.8	0.0	0.0	100.0	2.8	13.5	0.0	0.0
V10 (%)	100.0	0.5	2.6	0.0	0.0	100.0	0.0	2.0	0.0	0.0	100.0	0.0	4.2	0.0	0.0
V12 (%)	100.0	0.004	0.7	0.0	0.0	100.0	0.0	1.1	0.0	0.0	100.0	0.0	1.9	0.0	0.0
V24 (%)	40.4	0.0	0.0	0.0	0.0	12.9	0.0	0.0	0.0	0.0	39.8	0.0	0.0	0.0	0.0
D1 (Gy)	25.1	9.1	11.6	0.09	0.09	24.4	2.5	12.3	0.09	0.09	25.7	6.1	13.2	0.09	0.09
D10 (Gy)	24.7	5.9	6.3	0.09	0.09	24.0	0.6	0.1	0.09	0.09	25.1	2.8	0.1	0.09	0.09
D50 (Gy)	23.8	0.2	0.2	0.05	0.05	23.7	0.06	0.1	0.05	0.05	23.6	0.07	0.1	0.05	0.05
D95 (Gy)	20.5	0.01	0.03	0.01	0.01	22.3	0.01	<0.01	0.01	0.01	20.2	0.01	<0.01	0.01	0.01

PTV = planning target volume; PRV = spinal cord planning volume at risk.

**Table 2:** Dose calculation of thoracic metastases and risk organs for IMRT, carbon ion RT, and proton RT

	*IMRT*							*Carbon ion*							*Proton*						
	*PTV*	*Myelon*	*PRV*	*Right Lung*	*Left Lung*	*Heart*	*Esophagus*	*PTV*	*Myelon*	*PRV*	*Right Lung*	*Left Lung*	*Heart*	*Esophagus*	*PTV*	*Myelon*	*PRV*	*Right Lung*	*Left Lung*	*Heart*	*Esophagus*
Volume (cc)	30.2	58.5	99.7	1901.1	1719.4	553.5	93.4	30.2	58.5	99.7	1901.1	1719.4	553.5	93.4	30.2	58.5	99.7	1901.1	1719.4	553.5	93.4
Dmean (Gy)	23.7	0.5	0,52	1.3	1.1	0.14	1.5	23.3	0.44	0.6	0.23	0.02	<0.01	0.33	23.2	0.56	0.7	0.22	0.01	<0.01	0.08
Dmin (Gy)	14.9	0.0	0,00	0.02	0.04	0.03	0.06	13.1	0.0	0.0	0.0	0.0	0.0	0.0	13.8	0.0	0.0	0.0	0.0	0.0	0.0
Dmax (Gy)	25.5	15.5	15,12	22.7	15.3	0.8	10.2	25.7	12.4	16.6	21.5	8.5	0.09	8.9	26.9	12.6	16.2	20.9	7.4	1.6	15.6
V5 (%)	100.0	3.9	4,13	8.9	6.3	0.0	13.3	100.0	4.9	5.7	1.2	0.04	0.0	1.1	100.0	5.8	6.9	1.3	0.01	0.0	0.5
V10 (%)	100.0	0.8	0,81	1.8	0.4	0.0	0.05	100.0	0.5	1.8	0.4	0.0	0.0	0.0	100.0	0.6	2.1	0.5	0.0	0.0	0.1
V12 (%)	100.0	0.3	0,38	0.9	0.1	0.0	0.0	100.0	<0.01	0.7	0.2	0.0	0.0	0.0	100.0	0.02	0.8	0.3	0.0	0.0	0.05
V24 (%)	44.1	0.0	0,00	0.0	0.0	0.0	0.0	30.6	0.0	0.0	0.0	0.0	0.0	0.0	39.6	0.0	0.0	0.0	0.0	0.0	0.0
D1 (Gy)	25.1	9.4	9,55	11.9	8.2	0.5	8.9	25.2	9.4	11.3	5.6	0.4	0.09	5.2	25.9	9.5	11.6	6.3	0.09	0.09	2.2
D10(Gy)	24.6	0.6	0,63	4.5	3.7	0.3	5.6	24.4	0.09	0.06	0.5	0.1	0.09	1.0	25.1	0.5	0.07	0.2	0.1	0.1	0.1
D50 (Gy)	23.9	0.1	0,07	0.3	0.3	0.1	0.3	23.7	0.1	0.05	0.1	0.1	0.1	0.1	23.7	0.1	0.06	0.1	0.1	0.1	0.1
D95 (Gy)	22.0	0.01	0,01	0.04	0.03	0.01	0.03	20.3	<0.01	<0.01	<0.01	<0.01	<0.01	<0.01	18.9	<0.01	<0.01	<0.01	<0.01	<0.01	<0.01

PTV = planning target volume; PRV = spinal cord planning volume at risk.

**Table 3:** Dose calculation of lumbar metastases and risk organs for IMRT, carbon ion RT, and proton RT

	*IMRT*							*Carbon ion*							*Proton*						
	*PTV*	*Myelon*	*PRV*	*Intestine*	*Stomach*	*Kidney L*	*Kidney R*	*PTV*	*Myelon*	*PRV*	*Intestine*	*Stomach*	*Kidney L*	*Kidney R*	*PTV*	*Myelon*	*PRV*	*Intestine*	*Stomach*	*Kidney L*	*Kidney R*
Volume (cc)	54.7	9.9	18.6	3022.6	339.7	142.4	124.2	54.7	9.9	18.6	3022.6	339.7	142.4	124.2	54.7	9.9	18.6	3022.6	339.7	142.4	124.2
Dmean (Gy)	23.7	2.2	2.6	1.3	0.4	3.2	2.8	23.2	1.5	1.6	0.03	<0.01	<0.01	<0.01	23.2	2.5	2.6	<0.01	0.0	<0.01	<0.01
Dmin (Gy)	10.9	0.2	0.2	<0.01	0.1	0.3	0.2	10.9	0.0	0.0	0.0	0.0	0.0	0.0	10.9	0.0	0.0	0.0	0.0	0.0	0.0
Dmax (Gy)	26.2	10.7	12.6	13.4	6.3	9.5	11.1	27.4	9.3	10.6	1.8	0.6	<0.01	0.2	27.4	10.5	11.3	<0.01	0.0	<0.01	<0.01
V5 (%)	100.0	20.5	24.1	8.9	0.4	28.8	29.3	100.0	13.8	16.8	0.0	0.0	0.0	0.0	100.0	29.7	30.5	0.0	0.0	0.0	0.0
V10 (%)	100.0	0.2	3.2	0.3	0.0	0.0	0.7	100.0	0.0	2.0	0.0	0.0	0.0	0.0	100.0	0.05	1.4	0.0	0.0	0.0	0.0
V12 (%)	99.9	0.0	0.8	0.01	0.0	0.0	0.0	99.9	0.0	0.6	0.0	0.0	0.0	0.0	99.9	0.0	0.4	0.0	0.0	0.0	0.0
V24 (%)	46.9	0.0	0.0	0.0	0.3	0.0	0.0	19.4	0.0	0.0	0.0	0.0	0.0	0.0	23.7	0.0	0.0	0.0	0.0	0.0	0.0
D1 (Gy)	25.4	8.9	11.7	8.9	2.9	8.7	9.7	24.9	7.8	10.3	0.7	0.4	0.09	0.09	25.7	8.8	11.5	0.09	0.09	0.09	0.09
D10(Gy)	24.6	6.5	7.6	4.5	0.7	6.9	8.1	24.2	5.6	6.4	0.09	0.09	0.09	0.09	24.4	7.1	8.3	0.09	0.09	0.09	0.09
D50 (Gy)	23.9	0.9	1.0	0.3	0.3	2.5	0.6	23.7	0.1	0.1	0.05	0.05	0.05	0.05	23.6	0.6	0.7	0.05	0.05	0.05	0.05
D95 (Gy)	20.7	0.2	0.2	0.07	0.1	0.4	0.2	21.4	<0.01	<0.01	<0.01	<0.01	<0.01	<0.01	19.3	0.01	<0.01	<0.01	<0.01	<0.01	<0.01

PTV = planning target volume; PRV = spinal cord planning volume at risk.

## IV. DISCUSSION

Bone metastases are a very frequent secondary diagnosis associated with an advanced tumor disease, with the vertebral column being the most frequent location.^7^, ^22^, ^23^ The goal of local radiotherapy in the treatment of spinal tumors has been palliation of pain, prevention of pathologic fractures, and halting progression of or reversing neurologic compromise.^(24)^ Stereotactic body radiotherapy is being increasingly used in the management of metastatic spine tumors,^5^, ^13^ and may have a role in selected patients. A primary factor that limits radiation dose for local vertebral tumor control with conventional radiotherapy is the relatively low tolerance of the spinal cord to radiation.^(25)^ The role of radiation therapy in the treatment of metastatic tumors of the spine is well established and is often the initial treatment modality.^(26)^ Single‐fraction spinal stereotactic radiosurgery for metastases is both safe and clinically effective, allows a shorter treatment time, and better local control of the tumor with minimal risk of side effects.^(4)^

Our planning study compared single‐fraction spinal SBRT plans performed with step‐and‐shoot IMRT, carbon ion RT, and proton RT for cervical, thoracic, and lumbar bone metastases. First of all, we showed the feasibility of SBRT plans for proton and carbon ion RT. In all localizations, dose to the PTV did not show significant differences for the techniques. A retrospective study of 18 Gy single‐fraction SRS showed a local control of 92% with a median follow‐up of seven months (1–50 months), and neither neurological complications nor radiological abnormalities were recorded.^(27)^ Recent technological developments, including imaging technology for three‐dimension localization and pretreatment planning, the advent of intensity‐modulated radiated therapy, and a higher degree of accuracy in achieving target dose conformation while sparing normal surrounding tissue, have allowed clinicians to expand radiosurgery applications to treat malignant vertebral body lesions within close proximity to the spinal cord and cauda equina. This permits delivery of treatment plans with steep dose gradients between target volumes and adjacent organs at risk.^25^, ^28^ Spinal bone metastases are frequently located within the partial body with pedicle involvement; therefore, the PTV was defined according to this critical location next to the cord. The spinal cord is the dose‐limiting OAR for SRS. Our maximum doses at cervical and lumbar spinal cord in cervical and lumbar were 4.3 Gy and 9.3 Gy for carbon ion RT, as well as 8.1 Gy and 10.5 Gy for proton RT; dose to the spinal cord was considerably higher with IMRT (10.7 Gy–15.5 Gy). The maximum point dose for spine SBRT of 10 Gy is safe.^(29)^ Reports of myelopathy from stereotactic radiosurgery to spinal lesions appear rare (<1%) when the maximum spinal cord dose is limited to the equivalent of 13 Gy in a single fraction.^(22)^ The treatment times for particle RT were considerably shorter than with IMRT. Patients may be unable to maintain their position due to painful vertebral lesions, and longer treatment times are associated with a greater risk of motion.^(30)^ Our results showed satisfactory plans for all techniques; however, metastases located next to the myelin may benefit with particle RT, with a marginal benefit for carbon ion RT. A high dose 24 Gy SBRT still has limitations with regard to metastatic spinal cord compression. We acknowledge several limitations of planning studies, including ours: conclusions can be operator‐dependent and can also be dependent on choice of optimization parameters. In our study, a surrogate measure of treatment time is used and the time taken for patient setup and image guidance is not included. MRI fusion for spinal cord delineation and CT myelography has potential limitations. These uncertainties have led to our policy of limiting the dose within the spinal cord.

This planning study indicates that carbon ion and proton RT can deliver high‐quality PTV coverage for complex treatment volumes that surround the entire spinal cord. Large controlled trials are necessary to confirm these findings.
